# Supplementary material for: PepINVENT: generative peptide design beyond natural amino acids
Source: Chem Sci. 2025 Apr 16;16(20):8682–96. doi: 10.1039/d4sc07642g (PMC12002334; doi:10.1039/d4sc07642g)
Supplement: SC-016-D4SC07642G-s001 [file SC-016-D4SC07642G-s001.pdf]

Supplementary Information (SI) for Chemical Science.
This journal is © The Royal Society of Chemistry 2025

Supplementary Material

PepINVENT: Generative peptide design beyond the natural amino acids

Gökçe Geylan[ab*], Jon Paul Janet[a], Alessandro Tibo[a], Jiazhen He[a], Atanas Patronov[c], Mikhail Kabeshov[a], Werngard Czechtizky[d], Florian David[b], Ola Engkvist[ae] and Leonardo De Maria[d]

a. Molecular AI, Discovery Sciences, BioPharmaceuticals R&D, AstraZeneca, Gothenburg, Sweden.
b. Division of Systems and Synthetic Biology, Department of Life Sciences, Chalmers University of Technology, Gothenburg, Sweden.
c. Quantitative Biology, Discovery Sciences, BioPharmaceuticals R&D AstraZeneca, Gothenburg, Sweden
d. Medicinal Chemistry, Research and Early Development, Respiratory & Immunology, BioPharmaceuticals R&D, AstraZeneca, Gothenburg, Sweden
e. Department of Computer Science and Engineering, Chalmers University of Technology and University of Gothenburg, Gothenburg, Sweden
* Email: gokcegeylan96@gmail.com

Supplementary Information 1:

The additional information on the transformer model architecture trained for the peptide-based generative model is as follows:

- Encoder layers: six identical blocks consisting of a multi head self-attention and position-wise fully connected feed forward network were used.
- Decoder layers: similarly to the encoder layers, the decoder contains 6 layers. Each decoder layer has an additional sub-layer for multi-head attention on the encoder's output.
- Model dimension: the dimension of the input and output vectors was set to 256. This dimension is the same across all layers and serves as the size of the embeddings and the internal representations within the model.
- Number of attention heads: each multi-head attention mechanism is composed of 8 attention heads. Each attention layer splits the model dimension (256) into 8 subspaces of size 16.
- Dropout rate was set to 0.1.

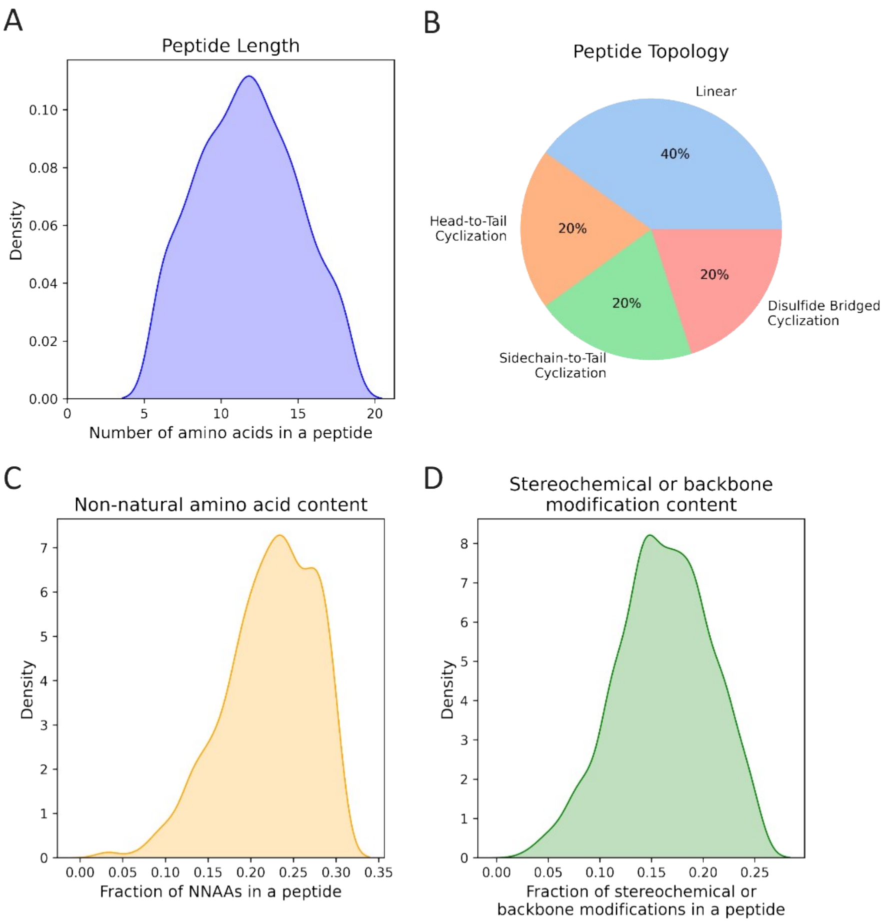

Supplementary Figure 1. The distributions of A) the peptide length, B) the topology distribution, C) the fraction of non-natural amino acids (NNAAs) and, D) the fraction of amino acids having N-methylated backbone or stereochemical modification.

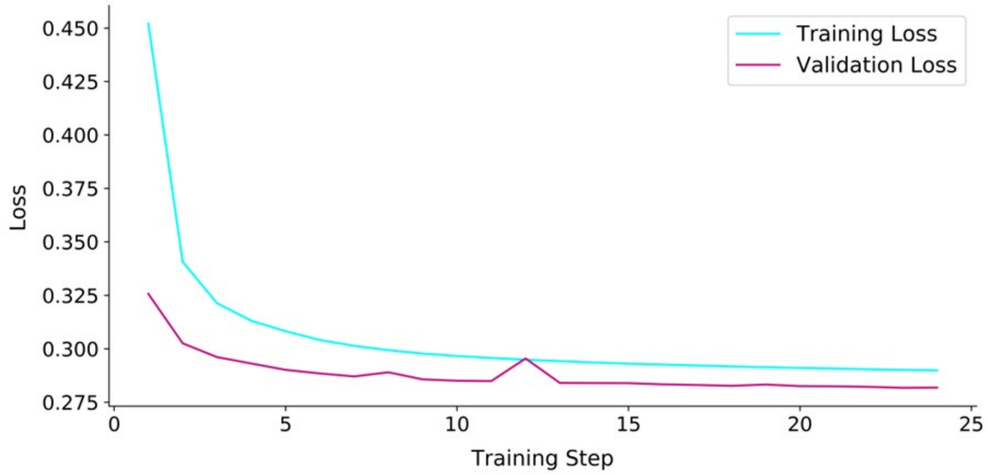

Supplementary Figure 2. The training and validation loss curves during the model training. The model state at the epoch 10 in which the losses plateau was selected as the final model.

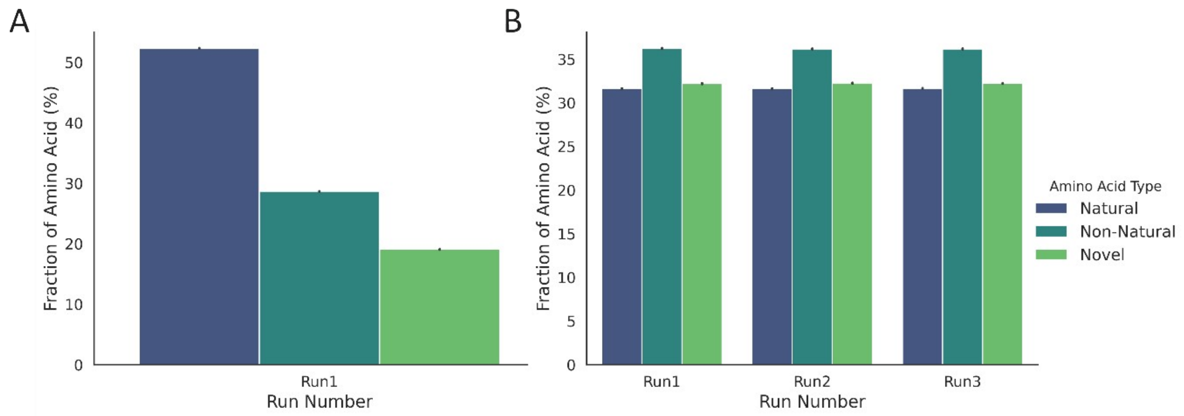

Supplementary Figure 3. The bar plot describing the percentage of the generated amino acid types in the test data peptides for A) beam search, and B) the triplicate runs for multinomial sampling. The plot shows the fraction of amino acids relative to the masked positions to showcase the occurrence of natural, non-natural and novel amino acids during the generative process.

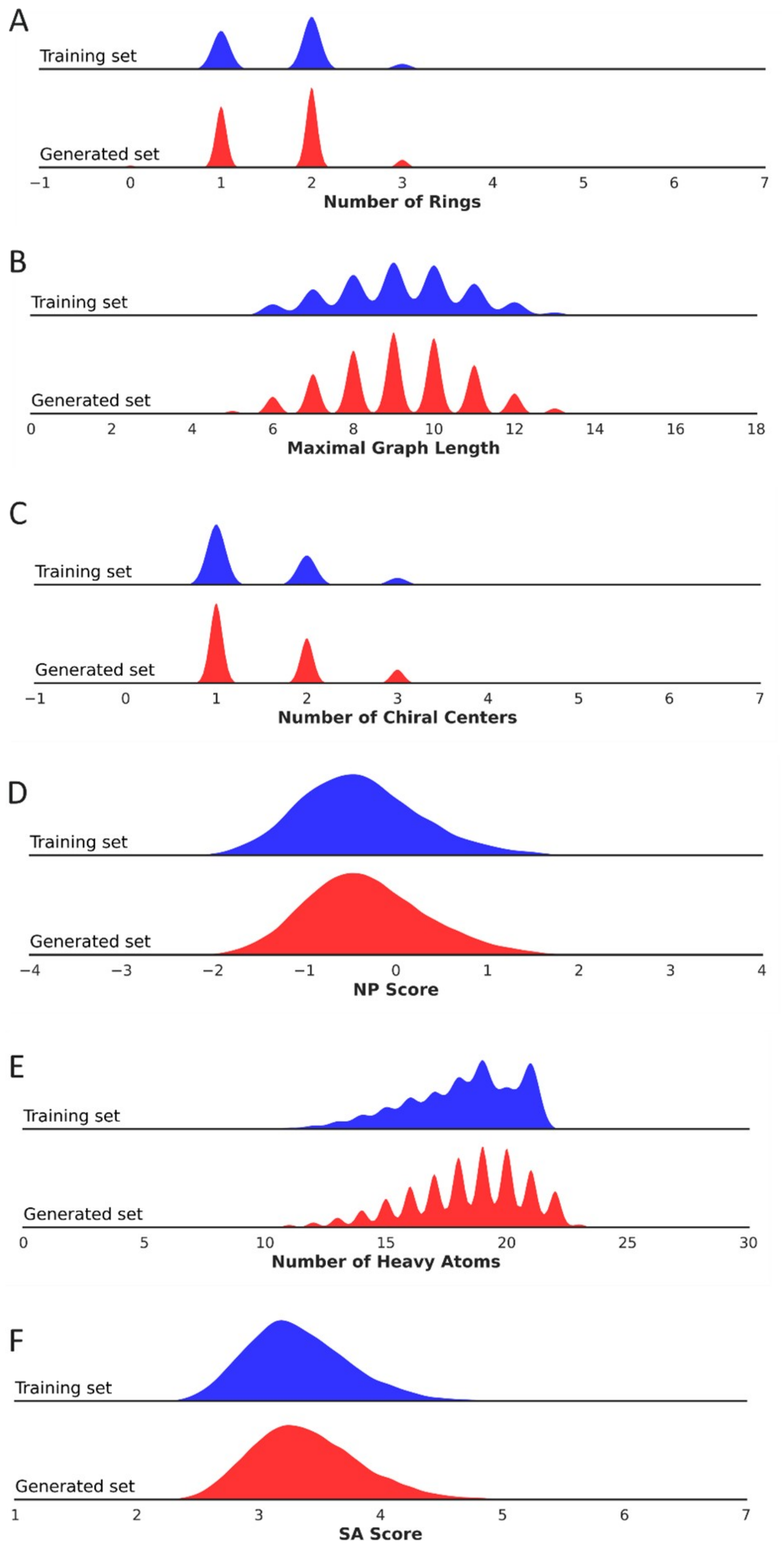

Supplementary Figure 4. The distributions of A) number of rings, B) maximal graph length, C) number of chiral centers, D) natural product-likeness score, E) number of heavy atoms, and F) synthetic accessibility score calculated on the building blocks from training set and novel building blocks generated during sampling.

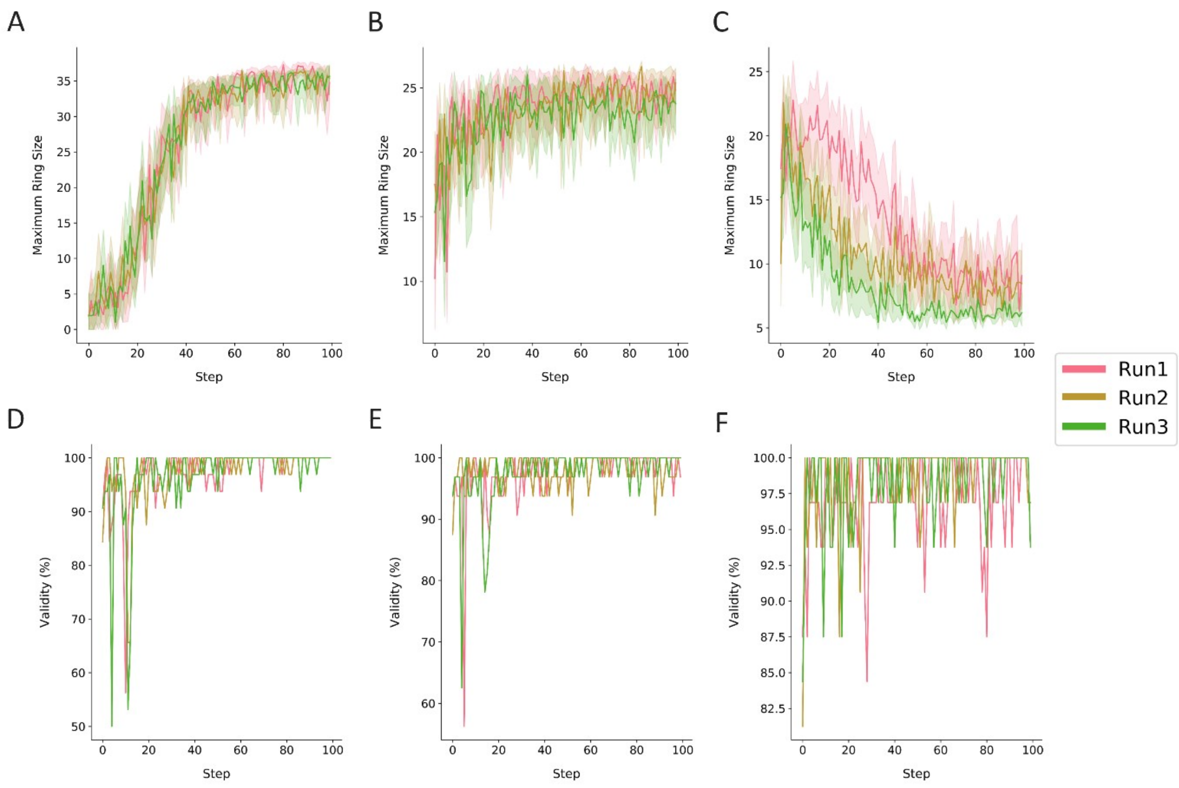

Supplementary Figure 5. The scoring component, the ring size of the largest ring, (A-C) and the percentage of valid peptides generated (D-F) over the learning steps in reinforcement learning (RL) runs for Scenario 1: Generating a peptide topology of interest. The evolution of the largest ring size and the validity of the generated peptides were plotted individually for the triplicate runs. The learning objective of A) maximizing the ring size and D) its validity, B) generating any macrocycle with an upper threshold of ring size to yield peptides with sidechain-to-tail or head-to-tail topologies E) its validity, C) minimizing the ring size and F) its validity. In every learning step, the average ring size of the batch was plotted with the 95% confidence interval.

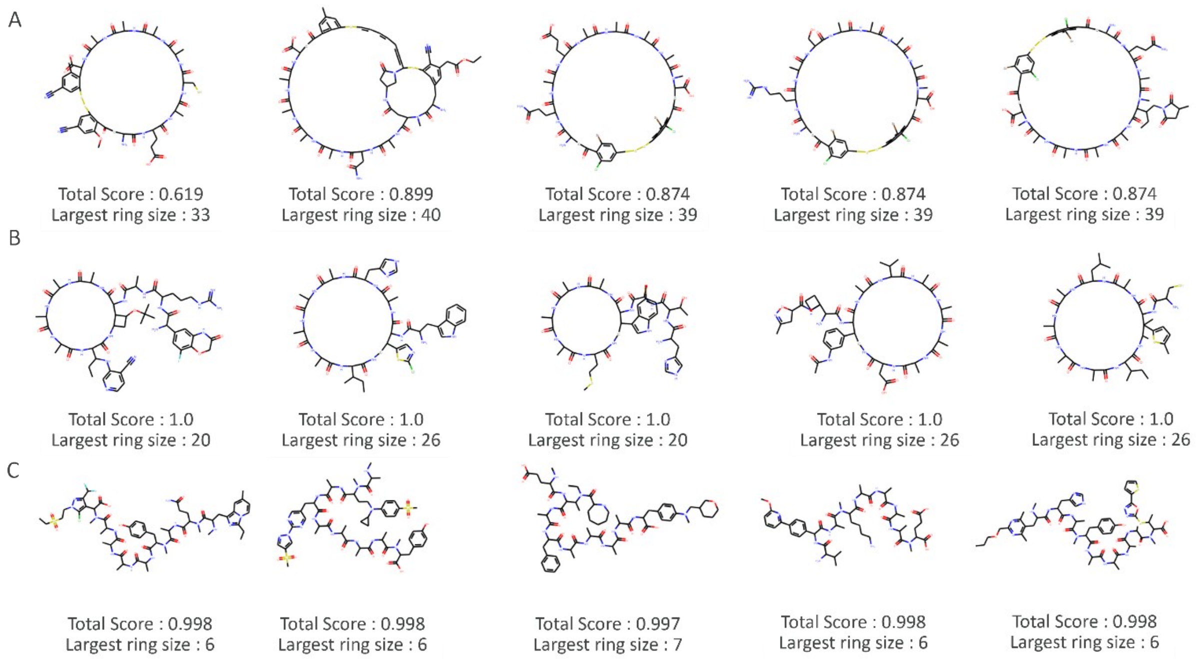

Supplementary Figure 6. Example peptides extracted from the last 10 steps of the RL runs that led to A) maximizing the ring size, B) preferring macrocycles with an upper limit of ring size, and C) minimizing the ring size, labelled with the total score and the largest ring size.

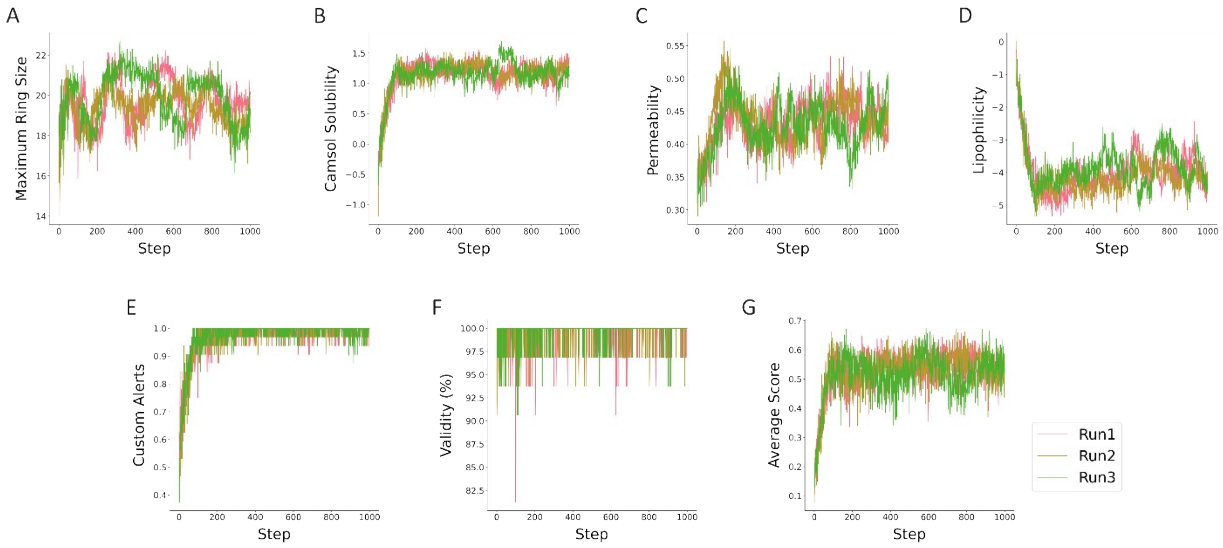

Supplementary Figure 7. The scoring components and the learning progress of the RL runs for Multi-Parameter Objective (MPO) task described in Scenario 2: Generating soluble and permeable cyclic peptides. The MPO task with the aim of designing soluble and permeable cyclic peptides were conducted in triplicate runs with multinomial sampling. The learning progresses of each triplicate were illustrated as A) the largest ring size, B) CAMSOL-PTM solubility score, C) the probability of belonging to the permeable class by the permeability predictor, D) the calculated lipophilicity score, E) the custom alerts score, F) the validity of the generated peptides and, G) the average score of the batch composed of all the scoring components of the MPO task.
